# The Involvement of Age, Gender, and Personality Variables in Alcohol Consumption during the Start of the COVID-19 Pandemic in Romanian University Students

**DOI:** 10.3390/bs13060519

**Published:** 2023-06-20

**Authors:** Cornelia Rada, Mihaela Lungu

**Affiliations:** 1 Biomedical Department, “Francisc I. Rainer” Institute of Anthropology, Romanian Academy, 050711 Bucharest, Romania; 2 Argeș County Centre for Educational Resources and Assistance, 110058 Pitești, Romania

**Keywords:** alcohol consumption, COVID-19 pandemic, personality, university students

## Abstract

This study aimed to explore the age, gender, and personality variables involved in alcohol consumption (AC) at the start of the COVID-19 pandemic in Romania among 210 bachelor’s and master’s students aged between 19 and 25 years. The results of the Freiburg Personality Inventory–Revised and the Alcohol Use Disorders Identification Test were examined using a logistic model and cluster analysis. The prevalence of problematic AC was relatively low (10.5%). The risk of males being part of the problematic AC cluster was 5.223 times higher than that of females (*p* < 0.001). Increasing age was associated with a decrease in the risk of belonging to the problematic cluster by a factor of 0.733 (*p* = 0.001). Increasing scores on the Frankness and Somatic Complaints personality scales were associated with a decreased risk of belonging to the problematic cluster of AC, with factors of 0.738 (95% CI, 0.643 to 0.848), Wald χ^2^(1) = 18.424, and *p* < 0.001 and 0.901 (95% CI, 0.813 to 0.999), Wald χ^2^(1) = 3.925, and *p* = 0.048, respectively. More action to prevent AC is needed in men, especially in those at the beginning of their university studies. It is necessary to intervene to decrease the interest in making a good impression (low scores on the Frankness Scale) so as to increase healthy autonomy using critical thinking and find a balance between the internal and external loci of control. Students from faculties with profiles that deal with health and its promotion are less vulnerable to problematic alcohol consumption, even if they have a withdrawn, pessimistic personality (low scores on Somatic Complaints).

## 1. Introduction

In many countries, the harmful consumption of alcohol by students has become a public health problem. Harmful use of alcohol and heavy episodic drinking have been cited as a cause of death [1] and have been associated with risk of driving without a seat belt, driving at unacceptable speed and involvement in accidents [2], increased risk of sexual assault [3], participating in other risky behaviors such as unprotected sex [4] and drug use [5], various diseases [6] and with decreased academic performance [7], and they have been associated with many other problems resulting in economic costs. Problematic alcohol consumption (AC) and repeated alcohol intoxication increase the risk of communicable diseases, infections, cancer, and other diseases as a result of immune system inhibition [8].

Some conceptual clarifications are needed regarding the definition of alcohol consumption according to the level of danger it represents. There are many forms of excessive drinking, such as high levels of drinking each day, repeated episodes of drinking to intoxication, drinking that is actually causing physical or mental harm, and drinking that has resulted in the person becoming dependent on or addicted to alcohol [9].

According to the World Health Organization (WHO) [10], these concepts are designated as follows: medium risk (drink too much on occasion); high risk–hazardous (drinking could lead to harm); and addiction likely–extremely hazardous (drinking is causing harm). “Heavy episodic drinking (drinkers only) is defined as the proportion of adult drinkers (15+ years) who have had at least 60 g or more of pure alcohol on at least one occasion in the past 30 days. A consumption of 60 g of pure alcohol corresponds approximately to 6 standard alcoholic drinks”. According to the American Society of Addiction Medicine [11], unhealthy use of alcohol is represented by “any use that increases the risk or likelihood of health consequences (hazardous use), or has already led to health consequences (harmful use)”.

The consumption of alcohol in large quantities upon entering university is practiced by some students as a rite of passage upon reaching this stage of maturity and independence [12,13,14]. However, certain nuances exist because maturity is not described in connection with the permission to consume alcohol, but also with having control over behaviors and being moderate [15].

The share of those who consume alcohol to the extent that it can harm their health differs from one study to another as a result of several factors, such as methodology, factors related to culture, tradition, legislation, etc. For example, in a study conducted in 17 Italian universities, data collected between 2018–2019, Messina et al. [16] found through AUDIT that over half were high-risk drinkers. In a cross-sectional study conducted between 2018 and 2019 on 722 medical students in Romania (Romanians and foreigners) aged 18–30, Nasui et al. [17] found dangerous AC in around 15%.

Studies examining unhealthy alcohol use in young people in relation to personality and psychological factors are fewer, and sometimes the results are different. Some examples below. Pre-pandemic studies have shown associations between certain personality traits and excessive alcohol consumption in the general population, such as impulsivity, thrill seeking, high scores on neuroticism and extraversion, and lower scores on conscientiousness and agreeableness [18,19].

Rada and Ispas in a study on 1359 young adults aged 18–30 years, found that AC was influenced by six accentuated personality traits: demonstrativeness, hyper-perseverance, uncontrollability, hyperthymia, cyclothymia, and exaltation [20].

In a systematic review of the literature among adolescents and young people, Adan, Forero, and Navarro found that binge drinking, or heavy episodic drinking, was associated with the following personality characteristics: high impulsivity and high sensation seeking, as well as anxiety, sensitivity, neuroticism (hopelessness), extraversion, and low conscientiousness [21].

In a study on 303 students in their first year from the Southeastern United States, Martin, Benca-Bachman, and Palmer found that high scores on the depression facet of the neuroticism domain of the NEO PI-R Personality Inventory were associated with greater AC. At the same time, an association was found between alcohol abuse and stress [22].

Schwarzbold et al. evaluated 707 medical students in Brazil in 2016 and found associations between risky alcohol consumption and high extraversion and low conscientiousness scores. At the same time, risky alcohol consumption was associated with a lower inhibition system and a higher fun-seeking activation system [23].

Griffin and Trull found that drinking alcohol in everyday life was associated with poor planning and deliberation [24]. Furthermore, Hakulinen and Jokela examined more than 39 thousand people on two occasions at an interval of approximately 5.6 years and concluded that long-term risky alcohol consumption had a statistically significant effect on personality as follows: it decreased emotional stability, agreeableness, and conscientiousness. Risky alcohol consumption was associated with increased extraversion, but the association was not consistent [25].

In the case of extraversion, which involves not only sociability and energy, but also hedonism and impulsivity, risky alcohol consumption could be explained by the high sensitivity to reward that motivates consumption to increase positive, euphoric states.

Another personality dimension to be taken into account is “Frankness” (or “openness”) found under this name in the Freiburg Personality Inventory–Revised (FPI-R) which will be analyzed in this study. The FPI-R attribute “Frankness” (or “openness”) refers to outgoing, unusual activities that are simple in nature. Lower scorers actively manage their impressions in an effort to leave a positive impression. Very few studies have been identified on this topic in relation to alcohol consumption. Personality trait Frankness, alcohol use, and brain response to stimulus are related. In their study Hoffmann et al. [26] found a correlation between error monitoring and error negativity (Ne or ERN) with personality FPI-R factors. The ERN was more pronounced for subjects scoring low on the “openness/frankness” scale, the “impulsiveness” scale, and the “emotionality” scale. Error-related negativity (ERN) is a large negative potential observed around 150 ms (usually between 60 and 120 ms) after an “incorrect” response in tasks that require “correct” identification of a stimulus presented and is thought to be generated in the Anterior Cingulate Cortex (ACC) area of the brain. ERN was found to be related to alcohol use; heavy drinkers displayed a smaller ERN amplitude [27]. In contrast with previous studies, ERN amplitudes were found to be higher for alcohol-dependent patients compared to healthy controls, particularly in patients with comorbid anxiety disorders [28]. Significant differences were found in 8 out of the 12 FPI-R instrument variables, between athletes and non-athletes [29]; athletes, most probably moderate alcohol users, had a higher average score on Frankness than non-athletes.

Several studies have examined alcohol use during the pandemic, most addressing anxiety, stress, and depression that may be involved in unhealthy drinking or other risky behaviors, but fewer have addressed the involvement of personality dimensions. Considering the above, this study aims to explore the age, gender, and personality variables involved in AC in young adulthood at the beginning of the COVID-19 lockdown. The research questions of this study are: what is the situation regarding alcohol consumption in the context of the beginning of the isolation imposed by the outbreak of the COVID-19 pandemic among university students? Does the profile of alcohol consumption change in this context imposed by social isolation? What personality factors are involved in problematic drinking? Could the personality dimension “Frankness” be a mediating factor in problematic alcohol consumption? Answering these research questions could provide useful information targeting interventions to prevent AC in college students, especially during times of crisis. At the same time, the involvement of personality dimensions in problematic alcohol consumption will be discussed through a questionnaire not used so far in this context.

## 2. Method

### 2.1. Participants, Evaluation Instruments

A total of 210 bachelor’s and master’s students (23% men and 77% women) at various universities in Romania, aged between 19 and 25 years (mean 21.08, median 21 years), participated in a larger study that dealt with the evaluation of psychological aspects and risky behaviors. The data were collected electronically between May and July 2020, when the first wave of the COVID-19 pandemic had set in, and students were participating in online courses. Throughout the research, a set of psychological questionnaires was used, namely: Strategic Coping Approach Scale (SACS) [30], Cognitive Emotion Regulation Questionnaire (CERQ) adapted for Romania by Perțe [31], the Depression, Anxiety, and Stress Scale (DASS-21) by Lovibond and Lovibond, adapted, standardized, and validated for the Romanian population by Perțe [32], and the Freiburg Personality Inventory–Revised (FPI-R) by Fahrenberg, Hampel and Selg validated for the Romanian population by Pitariu and Iliescu [33]. At the same time, an omnibus questionnaire was applied that covered demographic data, economic aspects, and family aspects, as well as containing the following topics: (a) relationships, sexual behavior, and sexuality; (b) physical activity; and (c) alcohol consumption; and The Alcohol Use Disorders Identification Test (AUDIT), developed by the World Health Organization to determine whether a person’s alcohol consumption may be harmful [1].

### 2.2. Materials

In this study, only the data obtained from the Freiburg Personality Inventory–Revised (FPI-R) and the Alcohol Use Disorders Identification Test (AUDIT) will be used. Therefore, the objectives of this present study are to identify the influence of age and gender on problematic AC and the relationships between AC and personality traits in the context of the beginning of the isolation imposed by the outbreak of the COVID-19 pandemic on Romanian students. The analysis was carried out using a logistic model. The alcohol consumption screening AUDIT questionnaire was designed to identify persons with hazardous and harmful patterns of alcohol consumption. Although hazardous drinking patterns are not associated with current disorder in the individual user, their identification is very important, because alcohol consumption at this level can be a risk factor for reaching harmful use (with consequences to physical and mental health) and alcohol dependence. After repeated alcohol consumption, a behavioral, cognitive, and psychological cluster is formed (alcohol dependence), from which it is very difficult to escape, and the costs of treatment and social recovery are very high [34]. Given that this study did not propose performing screening in the sense of AUDIT, but rather identifying in general the proportion of those with a consumption that could raise problems, cluster analysis was used. At the same time, because the sample contained only young adults, and because the last two categories—Hazardous and Extremely Hazardous—were poorly represented, with only two cases each, we decided to use latent class cluster classification, which was more appropriate for the analysis of data.

To assess AC, the 10 items from the Alcohol Use Disorders Identification Test (AUDIT) were used to evaluate AC in terms of quantity, frequency, and consequences on a Likert scale. The scores obtained for each item were collected and aggregated into a variable global quantitative indicator scale. For the latent class cluster model, each cluster contains a homogeneous group of persons (cases) who share common alcohol use behaviors, based on all 10 AUDIT items. The 10 items were analyzed via a cluster analysis technique using the specialized program Latent Gold [35]. According to the criterion BIC = 2670.39 and CAIC = 2722.39, the analysis in Latent Gold indicated a three-class model as being optimal. Cronbach alpha for the 10 items AUDIT questionnaire was α = 0.776. The *Akaike information* criterion, AIC = 2459.50 indicated a five-class best fit model, but with two classes sparsely populated, under 10%. Consequently, the more parsimonious model with three classes was preferred.

Subjects’ membership in a class was indicated by a categorical variable corresponding to three levels of AC description: *absent or limited*; *a moderate level*; and *a problematic, hazardous level*.

The Freiburg Personality Inventory (FPI-R, Freiburger Persönlichkeitsinventar), an omnibus assessment tool developed by Fahrenberg, Hampel, and Selg according to the multiphasic adult model, was used to assess personality dimensions. The questionnaire was chosen because it is of a multiphase type; in the description of each scale, there is a correspondence between several adjectives, which allows for a greater number of behavioral predictions.

The FPI-R test was adapted and validated for Romania in 2007. The national normative reference sample for Romania included 2400 subjects: 1200 women and 1200 men. For the 12 FPI-R personality measurement scales, including Life Satisfaction (LEB), Social Orientation (SOZ), Achievement Orientation (LEI), Inhibitedness (GEH), Excitability (ERR), Aggressiveness (AGGR), Strain (BEAN), Somatic Complaints (KORP), Health Concerns (GES), Frankness (OFF), Extraversion (E), and Emotionality (N), raw scores were calculated using the sum of the scores (0 = False or 1 = True) obtained from groups for 12 or 14 items associated with each scale, from among the 138 items indicated in the Freiburg test methodology. The Life Satisfaction scale consist of 12 items (α = 0.548), Social Orientation consist of 12 items (α = 0.523), Achievement Orientation scale consist of 12 items (α = 0.686), Inhibitedness scale consist of 12 items (α = 0.606), Excitability scale consist of 12 items (α = 0.748), Aggressiveness scale consist of 12 items (α = 0.737), Strain scale consist of 12 items (α = 0.769), Somatic Complaints scale consist of 12 items (α = 0.752), Health Concerns scale consist of 12 items (α = 0.606), Frankness scale consist of 12 items (α = 0.620), Extraversion scale consist of 14 items (α = 0.822), and Emotionality scale consist of 14 items (α = 0.736).

### 2.3. Procedure

The questionnaires were sent in editable PDF format. In order to preserve anonymity, each respondent received a code that was written down on the questionnaire. After completing them, they sent them to the coordinators, who were in charge of the collection. In the case of omissions or the misunderstanding of some answers, the questionnaires were sent back to the respondents (using their numeric code) for correction. All respondents provided written consent prior to participation and were informed that the European and national data processing standards were respected in each of the stages of the research. The research was performed in accordance with the Declaration of Helsinki in granting respect to human rights, and on the basis of an analysis of the informed consent questionnaire and the data collection procedure, the Ethics Committee approved the conduct of the research.

An ordinal logistic regression model [36] was used to identify the relationship between the scores observed on the 12 FPI-R personality measurement scales and membership in a cluster describing the AC category: absent or sporadic, moderate, and problematic.

The research hypothesis assumed that certain personality traits could predict the class of AC consumption. Two independent demographic variables were also entered for the ordinal logistic regression model: age and gender.

## 3. Results

Distribution of alcohol use risk categories, in accordance with the WHO, based on AUDIT scores, is presented in Table 1.

Cluster analysis of the AUDIT 10 items indicated the model with three clusters as being the optimal model for a minimum BIC = 2670.39. The formal name of each cluster was the result of the analysis of the response profile for the 10 items in the analysis. Table 2 presents the number of subjects assigned to each cluster as well as the mean score for AC.

Regarding FPI-R, the initial logistic ordinal model included all 12 scores on the FPI-R scales as independent variables, and analysis was performed in SPSS using the GENLIN and PLUM procedures. The final model included the demographic variables age and gender, and retained only two personality variables, for which significant effects were indicated through the B coefficients, namely: Frankness (OFF) and Somatic Complaints (KORP). For the final model, both Pearson and Deviance statistics, with a significance level of *p* > 0.05, indicated that the logistic model represented and fitted the observed data well. The χ^2^ test for parallel lines with a significance level of *p* > 0.05 indicated that the assumption of proportionality of odds was met, χ^2^(4) = 2.815, *p* = 0.589. No problems of multiple collinearities were identified when examining the VIF diagnostic statistics.

The significant effects of personality and demographics on AC, representing the B and EXP(B) values of the non-zero coefficients from the ordinary logistic regression equation, are presented in Table 2, along with the *p* level of significance (*p* < 0.05).

In Table 3, it can be seen that the risk of male persons belonging to the part of the reference cluster corresponding to “problematic” AC is 5.223 times higher than that of females (95% CI, 2.503 to 10.900), Wald χ^2^(1) = 19.396, *p* < 0.001). At the same time, an increase in age (expressed in years) is associated with a decrease in the risk of belonging to the “problematic” cluster, with a factor of 0.733 (95% CI, 0.606 to 0.887), Wald χ^2^(1) = 10.263, *p* = 0.001).

In addition, it was identified that increases in the scores on the Frankness and Somatic Complaints personality scales were associated with decreases in the risk of belonging to the “problematic” cluster, with factors of 0.738 (95% CI, 0.643 to 0.848), Wald χ^2^(1) = 18.424, *p* < 0.001 and 0.901 (95% CI, 0.813 to 0.999), Wald χ^2^(1) = 3.925, *p* = 0.048, respectively. In other words, the risk of belonging to the “problematic” cluster increases by 1/0.738 = 1.25 and 1/0.901 = 1.11, respectively, for each unit by which the scores on the Frankness and Somatic Complaints scales, respectively, decrease (Table 3).

As the problematic cluster was represented by 22 cases only, violating the rule of thumb requiring 10 events per predictor variable (EPV) for predictors in the above multivariate model, the results should be interpreted with caution, as indicated by Vittinghoff and McCulloch [37]. However, they also mentioned that systematically discounting results from models with five to nine EPV, particularly statistically significant associations, does not appear to be justified. In consequence, four univariate models, Gender, Age, OFF, and KORP, were also analyzed for each predictor. The B coefficients, *p* values, and odds ratios Exp(B) are presented in Table 4. The conservative univariate model assessments indicated regression results almost the same as those obtained using a multivariate model approach.

The multivariate model’s goodness of fit was improved by replacing the KORP variable with KORP2 = KORP × KORP, without affecting the interpretation of the results. The pseudo-R square statistic for the revised model increased from 0.148 to 0.151, and the corresponding p value for the KORP odds ratio decreased to 0.025 from 0.048.

## 4. Discussion

### 4.1. Prevalence of Alcohol Consumption

According to the AUDIT scores, only 2% consumed alcohol with high or extremely high risk, and 14.3% consumed alcohol with medium risk.

Analysis of the AUDIT items through the latent cluster model indicated that the prevalence of problematic AC (average score of AC above 13) was relatively low (10.5%), with most AC falling into moderate consumption (average score of consumption close to 5), very low consumption or non-drinkers (average score a little above 1). This overall result is comparable to that reported in other studies; however, the proportion of problem users differs from one study to another.

Compared to a study from the Netherlands on 5401 respondents from universities between the ages of 17 and 25 (the assessment was performed via AUDIT) [38] the present study found a lower percentage of heavy drinkers. Enstad et al. analyzed data from an Australian study with 786 subjects in late adolescence and young adulthood (18–25 years) and found, using AUDIT, that hazardous drinking was 38.9% [39]. In the present study, that percentage was lower.

The decrease in inhibition following AC caused officials during the pandemic to emphasize that in addition to social space, hygiene, and other steps to protect COVID-19, it was necessary to limit alcohol use. Some studies have indicated an increase in alcohol consumption during the pandemic [40,41].

Other studies have found that closing licensed places to AC and social distancing measures in response to the COVID-19 outbreak resulted in a decrease in harmful alcohol consumption [42,43].

In a study on students in the Southeastern United States conducted during the COVID-19 pandemic over different periods, Charles, Strong, Burns, Bullerjahn, and Serafine found that at the beginning of the pandemic, mood disorders, perceived stress, and AC were higher than before the pandemic, but that they had since returned to roughly pre-pandemic levels [44]. Research carried out during the enforcement of the “Lockdown” in Poland, at the initial stage, [45] found that some drank more, and others less, and in relatively similar proportions. It should be noted that those who drank less tended to be younger, and those who drank more had been heavy consumers even before the “Lockdown”.

Some studies have shown unchanged consumption [46,47,48]. Others have shown a decrease in consumption [49,50,51] that can be explained by both the fact that people were being careful about the money they spent, by the lack of social events, and by the ban on social events, club parties, and the decrease in peer pressure.

In 2019, the incidence of heavy drinking episodes at least once a month among the EU Member States was 19%, while in Romania, it was almost double that figure (comparable to Denmark and Luxembourg). Subject to the fact that data collection was carried out using other reporting methods and instruments, and on ages starting from 15 years old, it can nevertheless be stated that the prevalence of problematic alcohol consumption at the beginning of the pandemic among young students was relatively low [52].

It can be appreciated that the beginning of the pandemic did not generate dramatic changes among young people regarding alcohol consumption, probably because the use of social networks, accepted and used by the majority of young people, could partially compensate for social distancing. However, the effects of the increase in exposure to COVID-19 and the duration of isolation are to be considered in a future analysis.

### 4.2. Problematic Alcohol Consumption and Gender

It is necessary to make a clarification, namely that the answer variants of the questionnaire were only male, female. In the analyzed sample, the probability of men being part of the problematic AC cluster was higher than that of women. In a study of UK university students, Tarrant, Smith, Ball, Winlove, Gul, and Charles (2019) also found that males reported more AC than females [53]. Most studies indicate a higher share of excessive AC among men than among women [54,55].

Alcohol intoxication (drunkenness) is more common in men, but gender differences have begun to fade, especially in adolescents and young adults [56].

Völler, in their master’s thesis, which was carried out on a sample of 3671 Dutch citizens aged between 16 and 101, found a relatively stable consumption of alcohol by gender during the pandemic [57] which is similar to what was found in the present study on young Romanians.

### 4.3. Problematic Alcohol Consumption and Age

This study shows that the susceptibility of young people to peer influence regarding problematic AC decreases with increasing age. Here, a little more discussion is needed, because the results are not unanimous, and the definitions of adolescence vary [58]. The World Health Organization defines adolescence as the period between 10 and 19 years, but there are authors who place the upper limit at 26 years [59]. Just like Curtis [60] taking into account that during the period between 18 and 25 years of age the pubertal transition is completed, and a transition into professional, academic, and social responsibility is taking place, this period can rather be considered to be young adulthood.

The likelihood of adolescent alcohol use is influenced by peers and age group. Steinberg and Monahan, in a longitudinal and cross-sectional study of over 3600 males and females aged between 10 and 30 years, found that susceptibility to peer influences increases linearly between the ages of 14 and 18. This growth profile did not take shape between 10 and 14 years or between 18 and 30 years [61]. However, Duncan, Duncan, and Strycker found that with increasing age from 9 to 16 years of age, AC increased; however, both studies indicate that middle adolescence is the period at which resistance to peer pressure is lowest [62]. In the analyzed sample, which included students aged 19–25, this profile of alcohol consumption changes as a result of the maturity they are acquiring, such that conformity to the group norm decreases in intensity with increasing age. In addition, entering college at 18 or 19 years of age represents a transition period that in many situations involves changes regarding residence and social activities, and probably for this reason, the cluster with problematic AC is more heavily populated with students of this age than with those who are approaching completion of their master’s degree at 23–25. Bogowicz, Ferguson, Gilvarry et al. (2018), using the AUDIT on 1243 medical students, found a decrease in the proportion of students participating in problematic AC from the first year to the final year, where it was around 19% [63].

Different results were reported in the USA, where heavy alcohol use increases with age from 1.1% (16–17 years) to 4.2% (18–20 years) to 11.1% (21–25 years) [64]. This indicates that a critical point is the transition to the age of majority, which legally allows the sale of alcohol. Thus, entering another cycle of education or becoming a young adult can bring with it another negative aspect.

However, more exploration is needed to clarify why in the present study, as in others, problematic AC decreased slightly from 19 to 25 years. Such as, for example, what was observed by Lorant, Nicaise, Soto, et al. who found, in a study of 2700 Belgian students, namely that exposure to environmental factors in college (living on campus, living for a long time with many roommates) increased the risk of frequent and abusive AC, which can be explained by social involvement and the normative expectations of the group [65].

As a result, it can be stated that the beginning of the lockdown did not change the profile of excessive alcohol consumption according to age, with students from the younger age group being more numerous in the problematic alcohol consumption cluster.

### 4.4. Problematic Alcohol Consumption and Personality

In this study, higher scores on the Frankness scale decreased the risk of problem drinking. The Frankness scale was designed to distinguish between people who are oriented towards norms and rules and are interested in making a good impression on the one hand (low scores), from those who openly admit that they violate to a small extent certain social and conventional norms, and openly express their thoughts on the other (high scores) [33] (p. 118). The present study identified that those who were characterized by openness as a personality trait had a lower risk of belonging to the cluster with a high average score on AC. It is possible that these young people in the analyzed sample, being less conventional and less interested in making a good impression, did not respond to the pressure of the social group through excessive AC. In addition, the influence of the social group was very low, considering the social distancing imposed by the pandemic. Availability to be open, honest, relaxed, as a stable personality trait, can be a protective factor regarding alcohol consumption. At the other extreme, the tendency to conceal small weaknesses, insincerity, poor self-critical judgments, or even conceit, weak character (low scores) appears as a risk factor regarding alcohol consumption. In the present study it was identified that subjects with low scores on the Frankness scale are at risk for problematic alcohol, which is similar to what has been found in other studies [26,27,28]. Baudat et al., researched how teenagers manage information in their relationship with their parents and identified three types of information management strategies, namely Reserved, Communicators, and Deceptive [66]. They found that those in the Deceptive class were more likely to have problem drinking. Being frank is the exact opposite of self-hiding and this study shows that frankness is healthy. It is a more complex issue because honesty is learned, and it is to be expected that an authoritarian, critical, guilt-inducing style of education will decrease the honesty of young people, inhibit the capacity for self-disclosure. Carmo et al. found that authoritarian parenting can lead to the development of maladaptive perfectionism in children [67]. Hartman et al. found that self-concealment was associated with maternal authoritarianism and alcohol problems [68]. Mackinnon et al. also found that concealment of imperfection indirectly predicted alcohol problems [69]. Richardson et al. identified that self-critical perfectionism may be associated with alcohol use problems [70]. Perhaps the person consumes alcohol to feel more confident about reaching those unrealistic standards or to not feel persistent negative emotions.

The author of the instrument also made the scale to identify if the subjects give desirable answers, as a result it can be appreciated that these respondents did not tend to positively distort the answers.

Higher scores on the Somatic Complaints scale decreased the risk of problematic AC. The Somatic Complaints scale measures the degree to which people complain about somatic symptoms, with high scores indicating asthenic, sullen, withdrawn, pessimistic, and distrustful behavior, and low scores indicating sthenic, optimistic, and active behavior [33] (p. 115). The present study indicated that those who complained of depressive-type manifestations, asthenia, and physical symptoms had a lower risk of problematic AC. It is difficult to interpret this result, because similar results have not been found in other studies. For example, Wray, Dvorak, Hsia, Arens, and Schweinle found that optimism was negatively associated with alcohol consumption at age 14 [71]. Additionally, in a study of 3978 teenagers aged 14–18, Nguyen found that teenagers who were optimistic about their future were less likely to drink alcohol [72]. In the case of young adulthood in the present study, it is possible that this psychovegetative lability, this less positive evaluation of the state of health, this negativism that characterizes those who obtain higher scores on the Somatic Complaints scale, caused students to be more cautious regarding problematic AC. Miller, Fiellin, Rosenthal, and Saitz found that AC can lead to the onset of depressive symptoms and their worsening in those who already have them [73]. It is, therefore, possible that in the sample of students in the present study who tended to have an asthenic, depressive-type behavior, probably accentuated by the lack of social contact, were aware that this would be harmful to them and avoided drinking alcohol. In addition, it is another age period in which they were heading towards adulthood and did not tend to cheer themselves up by resorting to problematic AC. Another aspect that could be discussed as a future research hypothesis is the field in which the students were taking their university courses. Menon, Thamby, Jayaprakashan, Rani, Nair, Thennarasu, and Jaisoorya, in their study of 5442 college students in India, found that the lifetime prevalence of hazardous AC was lower in medical students [74]. In the present sample of Romanian students analyzed, over 50% of the respondents were enrolled in higher education in psychology, social assistance, physical education and sports, medicine, and biology: precisely those fields that are concerned with and promote healthy behaviors.

In a nationally representative online study of adults living in the United Kingdom, which was performed at three time points during the first part of the lockdown (March–July 2020) conducted by McBride et al., the authors found that those who explained the use of alcohol as being to cope with the situation generated by the pandemic were younger, consumed more alcohol before the pandemic, and presented elements of post-traumatic stress disorder and higher extraversion and death anxiety scores [75]. However, Völler’s study found that extraverts tended to drink more alcohol during the pandemic, but that this was not statistically significant, [57]. Additionally, in the present study, extraversion was not associated with problematic alcohol consumption.

Lui et al. carried out a review of the literature published from 2017 until March 2020. Extraversion—that is, excitement seeking—was correlated with alcohol use, while neuroticism—that is, impulsivity and angry hostility—was correlated with negative alcohol-related consequences. Low levels of conscientiousness and agreeableness were predictors of alcohol consumption, risky/hazardous drinking, and negative drinking-related consequences [76]. In the present study, aggressiveness and excitability had no effect on problematic alcohol consumption, which shows that this high degree of activity, spontaneity, reactivity, and dominance, and low degree of control, had no reason to have been activated as a result of social isolation. Therefore, the strength of the correlation between extraversion and alcohol consumption from other studies [77] needs to be analyzed not only on the basis of this pair, because high levels of conscientiousness, for example, could potentially reduce the strength of the relationship. More studies are needed to see the strength of the correlation between extraversion and alcohol consumption during periods of social isolation.

Sutin et al. in a longitudinal study of more than 7000 adults in the US, evaluated personality changes earlier, in 2020, and later, in 2021–2022, during the pandemic, and compared them to the pre-pandemic period, observing that at the beginning of the pandemic, only neuroticism decreased slightly, and only in the short term. After 2021–2022, neuroticism exhibited similar values as before, while at the same time, significant small changes were recorded in terms of lower scores for extraversion, openness, agreeableness, and conscientiousness [78]. This shows that the harmful changes at the level of personality appeared with the prolongation of the pandemic and the imposed restrictions.

The suspension of face-to-face participation in college courses and concerts, restrictions on meeting friends specific to young people, and even losing the jobs with which they paid for school, had no way of not resulting in changes to the students’ way of being. Even if, at the beginning of the pandemic, these changes regarding personality traits and problematic alcohol consumption did not seem dramatic, it can be stated that some classic traits associating alcohol consumption with personality have faded in terms of their manifestation, such as excitability, aggressiveness, extraversion, and emotionality (neuroticism). However, the isolation resulting from pandemic restrictions over a span of two years for children, adolescents, and young people has disrupted their transition into adulthood.

It cannot be said that these changes will be long-lasting; it is certain that the number of people requesting online therapeutic intervention during the period in which restrictions were operative increased a lot, which is an aspect that required difficult adaptation even among therapists [79,80].

After the restrictions had ended, another challenge was the reset, the return to the old routines before the outbreak of the pandemic, with some time being needed in order to reconnect and socialize.

## 5. Conclusions

The main limitation of the present study is the small sample size and the gender-imbalanced sample. In light of the results obtained regarding the involvement of personality in AC, additional studies are needed. Another drawback is that some of Cronbach’s alpha values go below 0.7, which is widely cited as the baseline for a trustworthy measuring scale. For greater comprehension and consistency, it is likely that some items on personality measurement scales need to be updated. However, a series of conclusions can be drawn.

During the restrictions imposed by the COVID-19 pandemic, which was generated by the SARS-CoV-2 virus, there were increases in anxiety, depression, and stress, especially at the beginning, when external social contact was almost prohibited. In this context, it was expected that some people would use AC as a resilience mechanism. However, in this study on Romanian students, the proportion of problematic AC was low. Less social contact, the restriction or prohibition of university courses that involved physical contact, moving from the university campus to live at home with parents, and the closing of clubs, bars, etc., are some of the potential causes of low AC. This fact shows that AC among young people has a socialization character, and as a result, control and legislation in this regard are necessary in universities. At the same time, it is possible that the field and specialization in which the young people are studying influences their consumption profile; in this case, when it is mainly about educational profiles, the mission is to promote physical and mental health.

The male Romanian students in this sample had a higher risk of problematic AC than the female students. As a result, the target group for prevention intervention remains male. However, the trend of increasing AC among girls and the decreasing gender gap in the last decade should not be lost sight of.

The beginning of the lockdown did not change the profile of excessive alcohol consumption according to age, with students from the younger age group being more numerous in the problematic alcohol consumption cluster. This research identified that the critical period for problematic AC occurs in the first years of entering college before following a slightly downward trajectory, meaning that there is a greater degree of vulnerability in this age group. As a result, more actions for preventing AC are necessary for this segment of freshman students who tend to celebrate their success upon entering college and begin to enter into friendships and erotic–sexual relationships on a different level than before, as AC can be interpreted as a rite of passage signifying the coming-of-age into adulthood. Therefore, preventive measures must be aimed at this 18–23-year-old age group, which comes with status challenges, especially since problematic AC in the pre-adult period can lead to alcohol addiction in the long term.

Only two personality scales from the Freiburg Personality Inventory–Revised were involved in the risk of belonging to the problematic cluster. Lower scores on the Somatic Complaints scale increased the risk of alcohol consumption. Therefore, the young Romanians in the analyzed sample who were characterized as being without psychosomatic problems, having healthy and active behaviors, being full of life, and rarely complaining of ailments had an increased risk of belonging to the cluster with problematic AC. It is necessary, through educational personal development interventions, to clarify this confusion whereby the feeling of well-being should be celebrated through excessive AC, especially since in the long term it will have negative consequences with respect to health.

Lower Frankness scale scores increased the risk for excessive AC. Therefore, the young Romanian students in the analyzed sample who were characterized by orientation towards norms and rules, and who were interested in making a good impression had an increased risk of belonging to the cluster with problematic AC. This distancing from dominant society and conformity with a possibly rebellious, heavy-drinking subgroup can create feelings of excitement and belonging. Educational interventions must be focused on increasing healthy autonomy, using critical thinking, and finding a balance between the internal and external loci of control, as well as diplomatically promoting a good life model.

Drinking alcohol could make a person talk more, tell the truth more easily, and make up stories. An idea emerges from this study, which of course should be explored in depth in the future, namely that people who have the personality trait of honesty are less likely to consume alcohol in order to have the courage to speak or tell the truth. Frankness is healthy and a protective factor against problematic alcohol consumption. Hypercriticism and perfectionism on the part of parents generates the feeling of not being good enough whatever you do and the anxiety of making mistakes can develop; in the struggle to hide the weaknesses, an emotional distress appears that the person can try to overcome by consuming alcohol. More attention should be given to these factors that may correlate with problematic alcohol use.

At the beginning of the restrictions imposed during the pandemic, the influence of personality factors on problematic alcohol consumption was low in both the present study and in other studies. However, it must be taken into account that some personality traits that have classically been associated with risky alcohol consumption (e.g., aggressiveness and excitability) have faded in manifestation. At the same time, the inconsistency of the results regarding the relationship between alcohol consumption and extraversion, both before and after, is necessary to understand in order to be able to consider other factors that mediate this association.

This study did not deal with psychological suffering (stress, anxiety, and depression) and coping mechanisms, but it can be stated that in the sample of young Romanian students, problematic alcohol consumption was not a mechanism for managing this completely new situation of restrictions.

## Figures and Tables

**Table 1:** Distribution of alcohol use risk categories based on AUDIT scores.

AUDIT Category WHO	Frequency	Percent	Valid Percent	Cumulative Percent
Low Risk (0–7)	176	83.8	83.8	83.8
Medium Risk (8–15)	30	14.3	14.3	98.1
Hazardous (high risk) (16–19)	2	1.0	1.0	99.0
Extremely Hazardous (addiction likely) (20–40)	2	1.0	1.0	100.0
Total	210	100.0	100.0	

**Table 2:** Clusters and average alcohol consumption score, according to AUDIT.

Cluster Name	N	%	Alcohol Consumption Mean Score
Absent or limited	89	42.4	1.27
Moderate	99	47.1	4.87
Problematic	22	10.5	13.27

**Table 3:** Demographic and personality effects on alcohol consumption.

Demographic and Personality Variables	B	*p*	Exp(B)
Gender (masculine)	1.653	0.000	5.223
Age	−0.310	0.001	0.733
Frankness (OFF)	−0.304	0.000	0.738
Somatic Complaints (KORP)	−0.104	0.048	0.901

**Table 4:** Effects of demographics and personality on alcohol consumption in univariate models.

Demographics and Personality Scales	B	*p*	Exp(B)
Gender (masculine)	1.183	0.000	3.265
Age	−0.248	0.003	0.780
OFF	−0.355	0.000	0.700
KORP	−0.100	0.027	0.904

## Data Availability

The database supporting the reported results is available only upon request due to confidentiality and ethical restrictions.

## References

[1] Hingson R., Zha W., Smyth D. (2017). Magnitude and Trends in Heavy Episodic Drinking, Alcohol-Impaired Driving, and Alcohol-Related Mortality and Overdose Hospitalizations Among Emerging Adults of College Ages 18–24 in the United States, 1998–2014. J. Stud. Alcohol Drugs.

[2] Williams R.D., Housman J.M., Woolsey C.L., Sather T.E. (2018). High-Risk Driving Behaviors among 12th Grade Students: Differences between Alcohol-Only and Alcohol Mixed with Energy Drink Users. Subst. Use Misuse.

[3] Muehlenhard C.L., Peterson Z., Humphreys T., Jozkowski K.N. (2017). Evaluating the one-in-five statistic: Women’s risk of sexual assault while in college. J. Sex Res..

[4] Woolf-King S.E., Fatch R., Cheng D.M., Muyindike W., Ngabirano C., Kekibiina A., Emenyonu N., Hahn J.A. (2018). Alcohol Use and Unprotected Sex Among HIV-Infected Ugandan Adults: Findings from an Event-Level Study. Arch. Sex. Behav..

[5] Colomer-Pérez N., Chover-Sierra E., Navarro-Martínez R., Andriusevičienė V., Vlachou E., Cauli O. (2019). Alcohol and Drug Use in European University Health Science Students: Relationship with Self-Care Ability. Int. J. Environ. Res. Public Health.

[6] World Health Organization (WHO) (2022). Alcohol. https://www.who.int/news-room/fact-sheets/detail/alcohol.

[7] Bernabéu Brotóns E., De la Peña Álvarez C. (2019). Cognitive Repercussions of Alcohol Consumption on Academic Performance at University: A Preliminary Study. Electron. J. Res. Educ. Psychol..

[8] Morojele N.K., Shenoi S.V., Shuper P.A., Braithwaite R.S., Rehm J. (2021). Alcohol Use and the Risk of Communicable Diseases. Nutrients.

[9] Babor T.F., Higgins-Biddle J.C., Saunders J.B., Monteiro M.G., World Health Organization (2001). AUDIT: The Alcohol Use Disorders Identification Test: Guidelines for Use in Primary Health Care.

[10] World Health Organization (WHO) (2022). Alcohol, Heavy Episodic Drinking (Drinkers Only) Past 30 Days. https://www.who.int/data/gho/indicator-metadata-registry/imr-details/458.

[11] O’Connor E.A., Perdue L.A., Senger C.A., Rushkin M., Patnode C.D., Bean S.I., Jonas D.E. (2018). Table 1, Unhealthy Alcohol Use: Terms and Definitions. Screening and Behavioral Counseling Interventions to Reduce Unhealthy Alcohol Use in Adolescents and Adults: An Updated Systematic Review for the U.S. Preventive Services Task Force [Internet].

[12] Crawford L.A., Novak K.B. (2006). Alcohol abuse as a rite of passage: The effect of beliefs about alcohol and the college experience on undergraduates’ drinking behaviors. J. Drug Educ..

[13] Brooks-Russell A., Simons-Morton B., Haynie D., Farhat T., Wang J. (2014). Longitudinal relationship between drinking with peers, descriptive norms, and adolescent alcohol use. Prev. Sci..

[14] Graupensperger S., Turrisi R., Jones D., Evans M.B. (2020). Associations Between Perceptions of Peer Group Drinking Norms and Students’ Alcohol Use Frequency Within College Sport Teams. Alcohol. Clin. Exp. Res..

[15] Månsson J., Samuelsson E., Törrönen J. (2022). Doing adulthood—Doing alcohol: What happens when the ‘sober generation’ grows up?. J. Youth Stud..

[16] Messina M.P., D’Angelo A., Ciccarelli R., Pisciotta F., Tramonte L., Fiore M., Ferraguti G., Vitali M., Ceccanti M. (2021). Knowledge and Practice towards Alcohol Consumption in a Sample of University Students. Int. J. Environ. Res. Public Health.

[17] Năsui B.A., Popa M., Buzoianu A.D., Pop A.L., Varlas V.N., Armean S.M., Popescu C.A. (2021). Alcohol Consumption and Behavioral Consequences in Romanian Medical University Students. Int. J. Environ. Res. Public Health.

[18] Stautz K., Cooper A. (2013). Impulsivity-related personality traits and adolescent alcohol use: A meta-analytic review. Clin. Psychol. Rev..

[19] Hakulinen C., Elovainio M., Batty G.D., Virtanen M., Kivimäki M., Jokela M. (2015). Personality and alcohol consumption: Pooled analysis of 72949 adults from eight cohort studies. Drug Alcohol Depend..

[20] Rada C., Ispas A.T. (2016). Alcohol consumption and accentuated personality traits among young adults in Romania: A cross-sectional study. Subst. Abus. Treat. Prev. Policy.

[21] Adan A., Forero D.A., Navarro J.F. (2017). Personality Traits Related to Binge Drinking: A Systematic Review. Front. Psychiatry.

[22] Martin K.P., Benca-Bachman C.E., Palmer R.H. (2021). Risk for alcohol use/misuse among entering college students: The role of personality and stress. Addict. Behav. Rep..

[23] Schwarzbold M.L., Haas G.M., Barni R.S., Biava P., Momo A.C., Dias T.M., Ayodele T.A., Diaz A.P., Vicente F. (2020). At-risk drinking and current cannabis use among medical students: A multivariable analysis of the role of personality traits. Braz. J. Psychiatry.

[24] Griffin S.A., Trull T.J. (2021). Alcohol use in daily life: Examining the role of trait and state impulsivity facets. Psychol. Addict. Behav..

[25] Hakulinen C., Jokela M. (2019). Alcohol use and personality trait change: Pooled analysis of six cohort studies. Psychol. Med..

[26] Hoffmann S., Wascher E., Falkenstein M. (2012). Personality and error monitoring: An update. Front. Hum. Neurosci..

[27] Bartholow B.D., Henry E.A., Lust S.A., Saults J.S., Wood P.K. (2012). Alcohol effects on performance monitoring and adjustment: Affect modulation and impairment of evaluative cognitive control. J. Abnorm. Psychol..

[28] Schellekens A.F.A., De Bruijn E.R.A., Van Lankveld C.A.A., Hulstijn W., Buitelaar J.K., De Jong C.A.J., Verkes R.J. (2010). Alcohol dependence and anxiety increase error-related brain activity. Addiction.

[29] Bara-Filho M.G., Ribeiro L.C.S., García F.G. (2005). Comparison of personality characteristics between high-level Brazilian athletes and non-athletes. Rev. Bras. Med. Esporte.

[30] Budău O., Albu M. (2010). Scala de Abordare Strategică a Copingului (SACS) [Strategic Coping Approach Scale (SACS)].

[31] Garnefski N., Kraaij V., Spinhoven P. (2010). (CERQ), [2002] Chestionarul de Evaluare a Copingului Cognitiv-Emoţional Adaptat și Standardizat pe Populația din România de Perțe, A. [Manual for the Use of the Cognitive Emotion Regulation Questionnaire, Adapted for Romania by Perțe, A.

[32] Lovibond S.H., Lovibond P.F. (2011). DASS, [1995], Manual Pentru Scalele de Depresie, Anxietate şi Stres: DASS 21-R, Adaptat în România de Perțe, A. Coord. Albu, M. DASS 21-R [DASS. Manual for the Depression Anxiety Stress Scales, School of Psychology, University of New South Wales, Sydney, Australia. Adapted for Romania by Perțe, A. Coord. Albu, M. DASS 21-R,].

[33] Fahrenberg J., Hampel R., Selg H. (2007). Freiburger Personlichkeitsinventar, [2001], Adaptat în România de Pitariu, H.P. și Iliescu, D. [Personality Inventory Freiburg Adapted for Romania by Pitariu, H.P. & Iliescu, D.].

[34] World Health Organization (1993). The ICD-10 Classification of Mental and Behavioural Disorders: Diagnostic Criteria for Research.

[35] Statistical Innovation (2021). Latent GOLD (Version 5.1). https://www.statisticalinnovations.com/.

[36] Garson G.D. (2016). Logistic Regression: Binary & Multinomial: 2016 Edition.

[37] Vittinghoff E., McCulloch C.E. (2007). Relaxing the rule of ten events per variable in logistic and Cox regression. Am. J. Epidemiol..

[38] Verhoog S., Dopmeijer J.M., De Jonge J.M., Van Der Heijde C.M., Vonk P., Bovens R.H., de Boer M., Hoekstra T., Kunst A.E., Wiers R.W. (2020). The Use of the Alcohol Use Disorders Identification Test–Consumption as an Indicator of Hazardous Alcohol Use among University Students. Eur. Addict. Res..

[39] Enstad F., Evans-Whipp T., Kjeldsen A., Toumbourou J.W., Von Soest T. (2019). Predicting hazardous drinking in late adolescence/young adulthood from early and excessive adolescent drinking—A longitudinal cross-national study of Norwegian and Australian adolescents. BMC Public Health.

[40] Graupensperger S., Fleming C.B., Jaffe A.E., Rhew I.C., Patrick M.E., Lee C.M. (2021). Changes in young adults’ alcohol and marijuana use, norms, and motives from before to during the COVID-19 pandemic. J. Adolesc. Health.

[41] Patrick M.E., Terry-McElrath Y.M., Miech R.A., Keyes K.M., Jager J., Schulenberg J.E. (2022). Alcohol use and the COVID-19 pandemic: Historical trends in drinking, contexts, and reasons for use among U.S. adults. Soc. Sci. Med..

[42] Callinan S., Smit K., Mojica-Perez Y., D’Aquino S., Moore D., Kuntsche E. (2021). Shifts in alcohol consumption during the COVID-19 pandemic: Early indications from Australia. Addiction.

[43] Glowacz F., Schmits E. (2020). Psychological distress during the COVID-19 lockdown: The young adults most at risk. Psychiatry Res..

[44] Charles N.E., Strong S.J., Burns L.C., Bullerjahn M.R., Serafine K.M. (2021). Increased mood disorder symptoms, perceived stress, and alcohol use among college students during the COVID-19 pandemic. Psychiatry Res..

[45] Chodkiewicz J., Talarowska M., Miniszewska J., Nawrocka N., Bilinski P. (2020). Alcohol Consumption Reported during the COVID-19 Pandemic: The Initial Stage. Int. J. Environ. Res. Public Health.

[46] Panagiotidis P., Rantis K., Holeva V., Parlapani E., Diakogiannis I. (2020). Changes in alcohol use habits in the general population, during the COVID-19 lockdown in Greece. Alcohol Alcohol..

[47] Anderson P., Llopis E.J., O’Donnell A., Kaner E. (2021). Impact of COVID-19 confinement on alcohol purchases in Great Britain: Controlled interrupted time-series analysis during the first half of 2020 compared with 2015–2018. Alcohol Alcohol..

[48] Bade R., Simpson B.S., Ghetia M., Nguyen L., White J.M., Gerber C. (2021). Changes in alcohol consumption associated with social distancing and self-isolation policies triggered by COVID-19 in South Australia: A wastewater analysis study. Addiction.

[49] Schmits E., Glowacz F. (2022). Changes in Alcohol Use During the COVID-19 Pandemic: Impact of the Lockdown Conditions and Mental Health Factors. Int. J. Ment. Health Addict..

[50] Gavurova B., Khouri S., Ivankova V., Kubak M. (2022). Changes in Alcohol Consumption and Determinants of Excessive Drinking During the COVID-19 Lockdown in the Slovak Republic. Front. Public Health.

[51] Ammar A., Brach M., Trabelsi K., Chtourou H., Boukhris O., Masmoudi L., Bouaziz B., Bentlage E., How D., Ahmed M. (2020). Effects of COVID-19 home confinement on eating behaviour and physical activity: Results of the ECLB-COVID19 international online survey. Nutrients.

[52] Eurostat (2021). One in Twelve Adults in the EU Consumes Alcohol Every Day. https://ec.europa.eu/eurostat/web/products-eurostat-news/-/edn-20210806-1.

[53] Wilsnack R.W., Wilsnack S.C., Kristjanson A.F., Vogeltanz-Holm N.D., Gmel G. (2009). Gender and alcohol consumption: Patterns from the multinational GENACIS project. Addiction.

[54] Salas-Wright C.P., Cano M., Hai A.H., Cano M., Oh S., Piñeros-Leaño M., Vaughn M.G. (2022). Alcohol abstinence and binge drinking: The intersections of language and gender among Hispanic adults in a national sample, 2002–2018. Soc. Psychiatry Psychiatr. Epidemiol..

[55] Bratberg G.H., Wilsnack S.C., Wilsnack R., Haugland S.H., Krokstad S., Sund E.R., Bjørngaard J.H. (2016). Gender differences and gender convergence in alcohol use over the past three decades (1984–2008), The HUNT Study, Norway. Public Health.

[56] Erol A., Karpyak V.M. (2015). Sex and gender-related differences in alcohol use and its consequences: Contemporary knowledge and future research considerations. Drug Alcohol Depend..

[57] Völler M. (2022). Changes in Alcohol Use in the Netherlands before and during the COVID-19 Pandemic: Exploring the Effects of Personality, Loneliness and Gender. Master’s Thesis.

[58] World Health Organization (WHO) (2022). Adolescent Health. https://www.who.int/health-topics/adolescent-health#tab=tab_1.

[59] American Psychological Association (APA) (2002). Developing Adolescents: A Reference for Professionals.

[60] Curtis A.C. (2015). Defining adolescence. J. Adolesc. Fam. Health.

[61] Steinberg L., Monahan K.C. (2007). Age differences in resistance to peer influence. Dev. Psychol..

[62] Duncan S.C., Duncan T.E., Strycker L.A. (2005). Alcohol use from ages 9 to 16: A cohort-sequential latent growth model. Drug Alcohol Depend..

[63] Bogowicz P., Ferguson J., Gilvarry E., Kamali F., Kaner E., Newbury-Birch D. (2018). Alcohol and other substance use among medical and law students at a UK university: A cross-sectional questionnaire survey. Postgrad. Med. J..

[64] Statista (2022). Current, Binge, and Heavy Alcohol Use in the United States in 2020. https://www.statista.com/statistics/354265/current-binge-heavy-alcohol-use-among-persons-in-the-us-by-age/.

[65] Lorant V., Nicaise P., Soto V.E., D’Hoore W. (2013). Alcohol drinking among college students: College responsibility for personal troubles. BMC Public Health.

[66] Baudat S., Mantzouranis G., Van Petegem S., Zimmermann G. (2022). How Do Adolescents Manage Information in the Relationship with Their Parents? A Latent Class Analysis of Disclosure, Keeping Secrets, and Lying. J. Youth Adolesc..

[67] Carmo C., Oliveira D., Brás M., Faísca L. (2021). The Influence of Parental Perfectionism and Parenting Styles on Child Perfectionism. Children.

[68] Hartman J.D., Patock-Peckham J.A., Corbin W.R., Gates J.R., Leeman R.F., Luk J.W., King K.M. (2015). Direct and indirect links between parenting styles, self-concealment (secrets), impaired control over drinking and alcohol-related outcomes. Addict. Behav..

[69] Mackinnon S.P., Ray C.M., Firth S.M., O’Connor R.M. (2019). Perfectionism, negative motives for drinking, and alcohol-related problems: A 21-day diary study. J. Res. Personal..

[70] Richardson C.M.E., Hoene T.H.M., Rigatti H.L. (2020). Self-critical perfectionism and daily drinking to cope with negative emotional experiences among college students. Personal. Individ. Differ..

[71] Wray T.B., Dvorak R., Hsia J.F., Arens A.M., Schweinle W.E. (2013). Optimism and Pessimism as Predictors of Alcohol Use Trajectories in Adolescence. J. Child Adolesc. Subst. Abus..

[72] Nguyen N.N. (2021). Optimism as a protective factor against alcohol use among Vietnamese teenagers. J. Subst. Use.

[73] Miller S.C., Fiellin D.A., Rosenthal R.N., Saitz R. (2019). American Society for Addiction Medicine (ASAM) Principles of Addiction Medicine.

[74] Menon P.G., Thamby A., Jayaprakashan K.P., Rani A., Nair B.S., Thennarasu K., Jaisoorya T.S. (2021). Does academic streams influence alcohol use in colleges?. Indian J. Psychiatry.

[75] McBride O., Bunting E., Harkin O., Butter S., Shevlin M., Murphy J., Mason L., Hartman T.K., McKay R., Hyland P. (2022). Testing both affordability-availability and psychological-coping mechanisms underlying changes in alcohol use during the COVID-19 pandemic. PLoS ONE.

[76] Lui P.P., Chmielewski M., Trujillo M., Morris J., Pigott T.D. (2022). Linking Big Five Personality Domains and Facets to Alcohol (Mis)Use: A Systematic Review and Meta-Analysis. Alcohol Alcohol..

[77] Sifuentes-Castro J.A., Lopez-Cisneros M.A., Guzmán-Facundo F.R., Telumbre-Terrero J.Y., Noh-Moo P.M. (2021). Personality traits and alcohol consumption in university students. Sanus.

[78] Sutin A.R., Stephan Y., Luchetti M., Aschwanden D., Lee J.H., Sesker A.A., Terracciano A. (2022). Differential personality change earlier and later in the coronavirus pandemic in a longitudinal sample of adults in the United States. PLoS ONE.

[79] Békés V., Aafjes-van Doorn K., Luo X., Prout T.A., Hoffman L. (2021). Psychotherapists’ Challenges with Online Therapy during COVID-19: Concerns about Connectedness Predict Therapists’ Negative View of Online Therapy and Its Perceived Efficacy Over Time. Front. Psychol..

[80] Singh S., Sagar R. (2022). Online Psychotherapy During the COVID-19 Pandemic: The Good, the Bad, and the Ugly. Indian J. Psychol. Med..

